# Are identities oral? Understanding ethnobotanical knowledge after Irish independence (1937–1939)

**DOI:** 10.1186/s13002-017-0189-0

**Published:** 2017-11-21

**Authors:** Fiona Shannon, Astrid Sasse, Helen Sheridan, Michael Heinrich

**Affiliations:** 1 School of Pharmacy and Pharmaceutical Sciences, Trinity College Dublin, Dublin, Ireland; 2 UCL School of Pharmacy, London, WC1N 1AX UK

**Keywords:** Ireland, Schools’ Folklore Scheme, Schools’ Manuscript Collection, Ethnomedicine, Ethnobotany, Oral identity

## Abstract

**Background:**

The Schools’ Folklore Scheme (1937–1939) was implemented at a pivotal time in Irelands’ political history. It resulted in a body of ethnological information that is unique in terms of when, why and how it was collected. This material consists of over 700,000 pages of information, including ethnomedicinal and ethnobotanical traditions, reflecting an oral identity that spans generations and that in many cases was not documented in writing until the 1930s. The intention of this study is to highlight the importance of the Schools’ Folklore Scheme and to demonstrate an ethnographic approach based on recollections of original participants of the scheme, to further understand the material in the collection and the impact it had on the participants.

**Methods:**

This study involves an analysis of both oral and archival data. Eleven semi-structured interviews with original participants of the scheme were carried out between April and September 2016. Their corresponding schools’ archival contributions to the scheme were located, and ethnomedicinal information was analysed and compared with the participants’ recollections.

**Results:**

The majority of participants’ stated the scheme had a positive impact on them. Five participants’ recalled collecting ethnomedicinal information, and there was a direct correlation between three of the participants’ ethnomedicinal recollections and their entries in the archives. One third of all the ethnomedicinal entries analysed included the use of a plant. There were 191 plant mentions and 64 plant species named.

**Conclusions:**

Contacting the original participants offers a novel approach of analysing this archival material. It provides a unique first-hand account of this historical initiative, an insight into how the scheme was implemented and how it impacted upon the children. The ethnomedicinal and ethnobotanical information provides an understanding of the medicinal practices in Ireland during the 1930s. The plant species that were both orally recalled by participants and documented in the archives are in keeping with key ethnomedicinal systems throughout the world.

**Electronic supplementary material:**

The online version of this article (10.1186/s13002-017-0189-0) contains supplementary material, which is available to authorized users.

## Background

Based on international agreements, most notably the Convention on Biological Diversity (1992), ‘States have, in accordance with the Charter of the United Nations and the principles of international law, the sovereign right to exploit their own resources pursuant to their own environmental policies, and the responsibility to ensure that activities within their jurisdiction or control do not cause damage to the environment of other States or of areas beyond the limits of national jurisdiction’ (CBD principle) [1]. In the discussions and processes leading to political agreements about biodiversity and their sustainable use, ‘the unique ways in which indigenous and traditional peoples perceive, use and manage their natural resources have been at the centre of the debates between representatives of nations, indigenous peoples, NGOs and international organisations. Specifically, it has been asked ‘how programs can be developed to guarantee the preservation and strengthening of indigenous communities and their traditional knowledge’ [2]. These major concerns are now commonly linked to the commercial opportunities arising from it (ecosystem services) and to the challenges posed by the rapid loss of biodiversity globally.

There is no universally agreed definition for traditional knowledge (TK). The World Intellectual Property Organisation (WIPO) has defined TK as ‘knowledge, know-how, skills and practices that are developed, sustained and passed on from generation to generation within a community, often forming part of its cultural or spiritual identity’ and comparably, the Convention of Biodiversity states that TK is ‘transmitted orally from generation to generation. It tends to be collectively owned and takes the form of stories, songs, folklore, proverbs, cultural values, beliefs, rituals, community laws, local languages, and agricultural practices, including the development of plant species and animal breeds’ [3]. One of the core aspects of TK is to recognise what is ‘traditional’ about traditional knowledge. It is accepted that ‘traditional’ is not related to the antiquity of the knowledge, but the way it is acquired and used. TK can be relatively new; however, it has a social meaning and legal character that is entirely unlike the knowledge in local communities acquired from migrants and industrialised societies [4].

Traditional medicinal knowledge (TMK) is one branch of TK that pertains to the maintenance of health, prevention, diagnosis and treatment of disease [3]. Throughout history, Irish people have relied heavily on TMK for their health care needs. This knowledge was shared among families and communities, or held by a specific person who would provide treatment for no monetary fee. Thus, it was considered to be a ‘people’s medicine’ and a fundamental aspect of Irish cultural history. It included ‘gifted’ people, sacred places, folk medicine (including plant and natural substances), prayers, charms, religious symbols and saints [5].

While biodiversity and their sustainable use have been widely recognised as important drivers of political actions, there also is the complex, but far less recognised question of cultural identities and their ‘construction’ [6]. The use of biodiversity be it as a food, a medicine or in any other way also allows a community to highlight their ‘uniqueness’ and ‘special characteristics’ as well as the complex interfaces with other cultures [6–8]. However, in the ethnopharmacological and ethnobiological debate, there is a gap with regard to research on how such cultural identities are constructed. Latin *identitat* and/or *identitas* is derived from *identidem*, a contraction of *idem et idem*, meaning ‘repeatedly’ (literally ‘same and same’) [7]. ‘Identity’ is both fluid and negotiated, and in our context, the core question relates to how the local and so-called ‘traditional’ uses of natural substances (plants, animals, fungi, food) contribute to the development of an ‘identity’. According to Noyes [8], ‘culture and identity become key concepts in global political struggles’ [6]. Here, we use the example of Ireland, only 15 years after independence. After many centuries of experiencing various levels of external political control, in 1921, Ireland signed a treaty that ended the war of independence and established the Irish Free State, a self-governing dominion within the British Commonwealth of Nations. The Irish language ‘Gaelic’ was a substantial feature of the politics that led up to this event [9], and the consequent nationalist policies that were implemented to reflect Irelands’ new national identity [10] were directly shaped by the attitudes of the key political figures towards Gaelic, as formed by ‘Conradh na Gaeilge’, the Gaelic League, a mass movement founded in 1893 with the aim of reviving the Irish language [9, 11]. One prominent figure involved in Irelands’ turbulent political history and in the rebuilding of an independent Ireland was Eamon De Valera, a charismatic and very powerful, but also controversial character [12]. He was involved in the 1916 Easter Uprising, the president of the Sinn Fein party from 1917 to 1926, the president of the first Dáil (Irish lower house) in 1919, one of the founders and first leader of the Fianna Fáil party from 1932 to 1959 and the President of Ireland from 1959 to 1973. Although opinions of de Valera’s political motives and actions have been deeply divisive among the Irish people, one legacy of his leadership is his contribution to the establishment of the Irish Folklore Commission in 1935, a unique organisation primarily devoted to the collecting of folklore around Ireland [13]. His views on the importance of Gaelic and Irish culture are frequently documented, and in one of his speeches at the opening of an exhibition on ‘Rural culture in Ireland’ in 1937, he spoke of the possibilities of this country with what he called ‘rural crafts’ as unequalled elsewhere from the point of view of the background of Celtic culture [14]. While embedded in a much wider development on rediscovering national identities in Europe [15–17], the Irish developments have been particularly well organised and are important nationally (see below).

### National Folklore Collection and Schools’ Folklore Scheme

The Folklore of Ireland Society was established in 1927 with the objective of collecting, preserving and publishing the folklore of Ireland. At that time, there was no existing collection of local (‘folk’) tradition in the country. Subsequently, under de Valera in 1935, the Irish Folklore Commission was formed, and as a result of its efforts, the National Folklore Collection at University College Dublin now holds one of the largest collections of oral and ethnological material in the world [18]. One of the Irish Folklore Commissions’ most successful initiatives was the implementation of the Schools’ Folklore Scheme (SFS) in 1937. It was a collaborative effort by the Department of Education, the Irish National Teachers’ Organisation and the Irish Folklore Commission that involved collecting and documenting traditions of the Irish people by the agency of senior primary school children, aged 11 to 14. It included 5000 primary schools, over 100,000 students and resulted in approximately 739,000 pages of information of folklore and Irish traditions, including the use of plants and other natural substances as ‘cures’. During these periods, primary school children collected material from older people in their local communities and wrote their findings in small copybooks (SC). Following this, a portion of material was transcribed to large manuscripts (LM) that were issued to each school by the Department of Education. All of the large manuscripts (451,000 pages in total) were then returned to the National Folklore Collection and compiled into 1128 volumes, forming the Schools’ Manuscript Collection (SMC). Additionally, many of the schools forwarded a selection of the children’s small copybooks (288,000 pages in total) to the National Folklore Collection, where they were then boxed and stored [19]. The sheer quantity and diversity of information located in the archives pose challenges for its analysis. In the presented research, we are using a novel perspective, combining approaches from oral ethnohistory and ethnopharmacology.

### ‘Capturing traditional Irish knowledge’—Schools’ Folklore Scheme

The material collected in the Schools’ Folklore Scheme provides a huge repository of historic Irish local knowledge, which potentially offers unique insights into the twentieth century’s ethnomedicinal and ethnobotanical history. Much of the information had never been recorded in the written form, and over 18% of the contributions are in Gaelic ‘personal communication with Anna Bale in the National Folklore Collection’. The material was collected at a pivotal time in Irelands’ political history and at a time when the majority of the population lived in poverty. Emigration was devastating rural communities, there was no influence from television (introduced 1949) and the use of radio was only in its infancy.

As of 2016/2017, the documents in the SMC could still be linked to the very last remaining survivors, who participated in the survey and who are in their late 80s and early 90s, offering the last opportunity to record their experience as an element of Irish oral history. The current study captures the voices and recollections of 11 living participants to the study and links them back to their specific school’s contributions in the archives, offering context and a different perspective on the collection itself, and the ethnomedicinal knowledge housed therein. Each oral contribution can be viewed as a ‘recreation of tradition’ as proposed by Tonkin [20]. Validity, reliability and bias of oral sources can be of concern. In this case, the oral accounts are removed some 80 years from the original events; however, this should not be considered a flaw, but rather as proposed by Tosh [21] and Portelli [22], the reliability of the accounts lies in the subjectivity of the contributors. The method of collecting ethnobotanical information retrospectively has also been demonstrated in Poland in a study investigating wild food plants consumed by respondents when they were children. These data were then compared to archival material collected in a 1948 study that was implemented by the Polish Folklore Society, and further studies carried out from 1964 to 69 and 2000–2003 by young ethnographers and ethnography students. However, the participants were not linked to the original study which had been completed by a range of individuals (including children) [23].

To date, there are limited published studies based on the material in the SMC, no studies on the material in the SC and no studies with the original participants of the scheme in a current setting. Briody’s 2007 book *The Irish Folklore Commission 1935–1970 history, ideology, methodology* offers a comprehensive insight into the history and implementation of the Schools’ Folklore Scheme. Other research that utilised material on TMK in the SMC include Allen and Hatfield’s 2004 book *Medicinal Plants in Folk Tradition, An ethnobotany of Britain and Ireland* [24] and a study conducted by Dolan [5] that refers to the TMK documented in the SMC as part of the background research for the article on the use of medicinal plants in the South West of Ireland.

### Research question

The information archived in the Schools’ Manuscript Collection can be analysed in a variety of ways. This is a direct consequence of how the archives are indexed and how information is stored together with the nature of the TMK documented within. Analyses can be directed by location (e.g. school, county, geographical area), type of treatment used (e.g. plant, food, religious), modality of treatment (e.g. externally applied, internally taken) and disease classification (e.g. dermatological, respiratory) or others. Although exploring the TMK information through such categories offers insight into the material in the archives, the unique approach taken in this study enables a more enriched understanding, viewing the material through a new lens. In capturing the oral recollections of living participants of the scheme, the archives are viewed with a different perspective adding a new depth to the analyses. The research question we present is:

*Can the oral recollections from individual contributors to the Schools Folklore Scheme, captured eighty years after the event, contribute to our understanding of the study design, clarify the importance of material collected, reveal potential bias of teachers in selection of content and identify any perceived benefits that the contributors to this study may have gained by participating in this historic initiative, which took place only 15 years after Irish Independence?*

## Methods

### Research area

In 1930s Ireland, the cultural, political and ideological background governed the formation of the National Folklore Collection. This was directly linked to the decline of the Irish language and a sense of urgency with regard to salvaging and recording the oral traditions of Gaelic Ireland. This was described by Briody as a ‘last minute effort to save as much of the riches of Irish folklore for posterity before they were irretrievably lost’ [13]. During this period, Ireland was ‘economically depressed’, and the majority of the Irish people lived in poverty [25]. In 1936, the total population was just under 3,000,000. The Schools’ Folklore Scheme follows ideas developed in the late nineteenth century most notably in Estonia and in the Baltic states and Scandinavia. It represents the largest folklore collection ever implemented globally. Its origins are derived from a combination of native and foreign influences, specifically the Baltic states and Scandinavia. In 1923, Énrí Ó Muirgheasa, a teacher and collector for the Folklore of Ireland Society, proposed a scheme to the Department of Education that involved distributing notebooks to primary school teachers across the country and requesting that they collect and document information on Irish folklore. This scheme was implemented in 1933. However, due to lack of planning and poor participation among teachers, the results were not successful. In 1928, another collector named Séamus Ó Duilearga travelled to Northern Europe on a study trip, where ‘the science of folklore was most developed’. During this trip, while in Sweden, Ó Duilearga was introduced to the concept and importance of a systematic collection of folklore as well as the method of collecting material through primary school children [13]. Since the nineteenth century, Estonia has also actively participated in systematic folklore collection, and as a result, the Estonian Folklore Archives contain an extensive amount of ethnographic data, including data on medicinal cures. These collections were initiated by the pharmacist Johann Georg Noel Dragendorff (1836–1898) who made a first appeal to collect ethnobotanical folklore in 1877 [26]. Interestingly and in the 1920s and 1930s prior to and at the time of the Schools’ Folklore Scheme, an Estonian school teacher Gustav Vilbaste (1885–1967) used school children for the collection of ethnobotanical data [27]. In the 1930s, the Polish ethnologist Adam Fischer (1889–1943) collected ethnobotanical data in the Ukraine [28].

The Schools’ Folklore Scheme was successfully implemented in 1937. A booklet that contained hundreds of questions and suggestions for compiling information on a wide range of folkloristic and ethnological topics, arranged under 55 separate headings, including ‘disease, healing and herbs’ was prepared and distributed to teachers taking part in the scheme, across the country. During the school period of September 1937 to June 1938, the time allotted to Irish and English composition for pupils in fifth and higher standards was devoted to folklore composition and the recording of stories and traditions collected by the pupils from their homes and districts. Each week, the teacher chose one specific heading from the supplied booklet, read out the questions and transcribed them on the blackboard. The children gathered information and documented it in their small copybooks as part of their homework. The teacher then selected information to be transcribed into the large manuscripts by a child considered to have the best handwriting [13].

The 1930s study in Ireland drew from a Gaelic culture that was heavily influenced by oral tradition, with storytelling embedded in the Irish cultural life tracing back to the pre-Christian era when ‘Druids or priests’ and ‘filidh or poets’ were responsible for the transmission of a community’s knowledge [29].

**Fig. 1:**
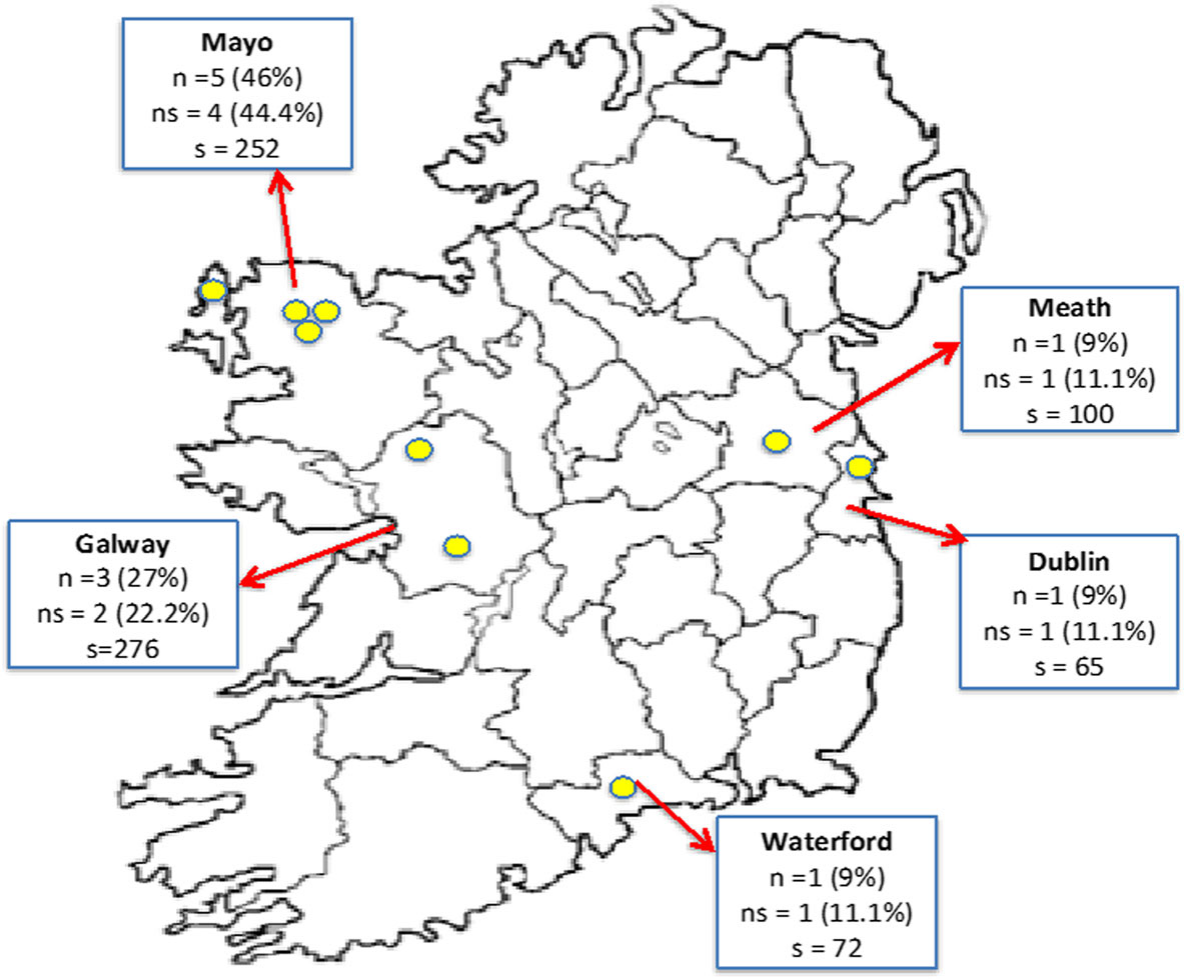
Ireland map—participants’ schools and total schools that participated in the scheme within those counties

The participants in the current study were educated during this era in counties, Dublin, Meath, Waterford, Galway and Mayo (Fig. 1). In 1936, counties Dublin, Meath and Waterford, located on the eastern side of the country, had populations of 586,925 (19.8%), 61,405 (2.1%) and 77,614 (2.6%), respectively, and counties Galway and Mayo, located on the western side of the country had populations of 168,198 (5.7%) and 161,349 (5.4%), respectively [30]. One participant attended school in the parish of Swords, North County Dublin. Dublin, the largest city and capital of the ‘New Free State’, was in an area known as ‘the Pale’, where Ireland had been under British Rule in the Middle Ages. One participant attended school in the parish of Dunshaughlin in County Meath, an agricultural county that had the most fertile soil in the country and was located on the perimeter of ‘the Pale’. One participant attended school in the parish of Camphire, County Waterford, located south of ‘the Pale’ a county that benefited economically from Waterford Port, the principal sea link from Ireland to the mainland of Europe. Four participants attended three different schools across County Mayo in the parishes of Ballina, Crossmolina and Belmullet. Three participants attended two schools in the parishes of Dunmore and Loughrea in County Galway. Counties Galway and Mayo are a greater distance from ‘the Pale’ on the west of Ireland. The majority of ‘Gaeltacht’—Irish-speaking regions—were located in these counties, and the quality of the land was relatively poor in comparison to the rest of the country. Although the economy was in crisis and poverty was a major issue in the 1930s, ‘The School Attendance Act’ implemented in 1926 required every child to attend school from 6 to 14 years of age [31]. This resulted in a relatively high attendance rate of 78% of children attending primary school in 1936 [32].

### Ethnological interviews

A snowball sampling method as described by Vogt [33] was implemented to contact original contributors to the scheme via social media, pharmacies, religious institutions, family and friends, posting information on notice boards and contacts from the National Folklore Collection in UCD. After extensive inquiries, 11 participants of the 1930s scheme were located and agreed to take part in an ethnological survey (Table 1).

**Table 1:** Participants’ information

Participants	School location	Participant sex	Age at time of interview
P1	Dublin	F	89
P2	Mayo	F	91
P3	Meath	M	92
P4	Mayo	F	89
P5	Galway	F	90
P6	Waterford	F	91
P7	Mayo	F	86
P8	Mayo	F	90
P9	Mayo	F	89
P10	Galway	F	93
P11	Galway	F	92

This cohort comprised of ten females and one male participant. Five interviews were secured through a wider network of friends and family, four were referred by the National Folklore Collection, one was contacted through a pharmacy and one through an online notice board in Trinity College Dublin. Two further interviews were arranged but were cancelled due to ill health of the potential participants. Interviews took place in a variety of locations across Ireland, and the questionnaire was completed in Boston, USA. The structured interviews were audio recorded and ranged from 25 to 50 min in length. In addition to documenting the oral recollections, the participant’s corresponding school’s SC and LM written material was located in the archive and their personal contributions were identified, where possible. A combination of oral and archival data is examined in this study; the data documented from 11 interviews with original participants and their corresponding schools’ SC and LM material. All interviews took place between April and September 2016 in the form of structured interviews/questionnaire. It was designed to cover a range of areas as outlined in Table 2. During face-to-face interviews, time was allowed for the elaboration of topics as directed by responses and the interviews did not all follow the same sequence of questions.

**Table 2:** Outline of discussion topics and questions used in interviews

General topics	Details documented
Informant information	Age, date of birth, place of birth.
School details	Name, location, religious ethos, sex of teacher.
General conversation	School and classroom description, number of pupils in school and class, daily class routine.
The Schools’ Folklore Scheme	When and how they heard about the scheme, how the scheme was implemented, what part it played in the school curriculum, how did the teacher advise on collecting the information, whom did they collect information from, how often did it happen, how was the information recorded, did they think the scheme had a positive or negative affect on them, would they recommend a comparable scheme be implemented today.
Traditional medicinal knowledge	Did they collect and record TMK, what specific TMK can they recall, did they pass this information on to younger generations.

### Schools’ Manuscript Collection and small copybook archival data

All of the information obtained from the Schools’ Folklore Scheme is now archived and stored in the National Folklore Collection at University College Dublin, Ireland. The large manuscripts are bound together and stored in 1128 separate volumes, and as of 2016, these volumes are digitised and available to view online at duchás.ie. They are easily accessed by an index system that labels them by volume number, school name, barony, parish, teacher name and corresponding SC (when available). A subsequent index exists for the LM listing the TK categories that are located in each volume and the corresponding pages, enabling easy access to all TMK information. However, there is no index system for the SC. As a result, in order to locate TMK information, all pages in each copybook must be viewed. The information obtained from the ethnological surveys, i.e. school name, teacher name and location were cross-referenced with the National Folklore Collection index system to locate the participants’ corresponding school’s LM material and SC if available. The LM index system was utilised to identify each schools’ TMK information, and the SC were analysed to locate and transcribe all TMK.

### Disease, healing and herbs

‘Folk healing’ is one narrative that is handed down via oral communication and socialisation. It is tied to ecology, geography and local culture, based on ‘traditional beliefs’. As there are as many different folk beliefs and practices and health systems, as there are folk cultures in the world [34], for the purpose of this paper, we will not attempt to analyse all the varying definitions applied to ‘folk healing’ or ‘folk medicine’. Instead, we have classified all the ‘disease, healing and herbs’ data collated from the archives and interviews in this study under the domain of traditional medicinal knowledge (TMK).

### Plant species

The ‘plant’ names that are documented in the SC, LM and from the interviews are in vernacular form. Utilising the Irish plant book *Ireland’s Generous Nature* [35] and the online checklist www.theplantlist.org, the most probable plant species was identified where possible.

### Statistical analysis

**Fig. 2:**
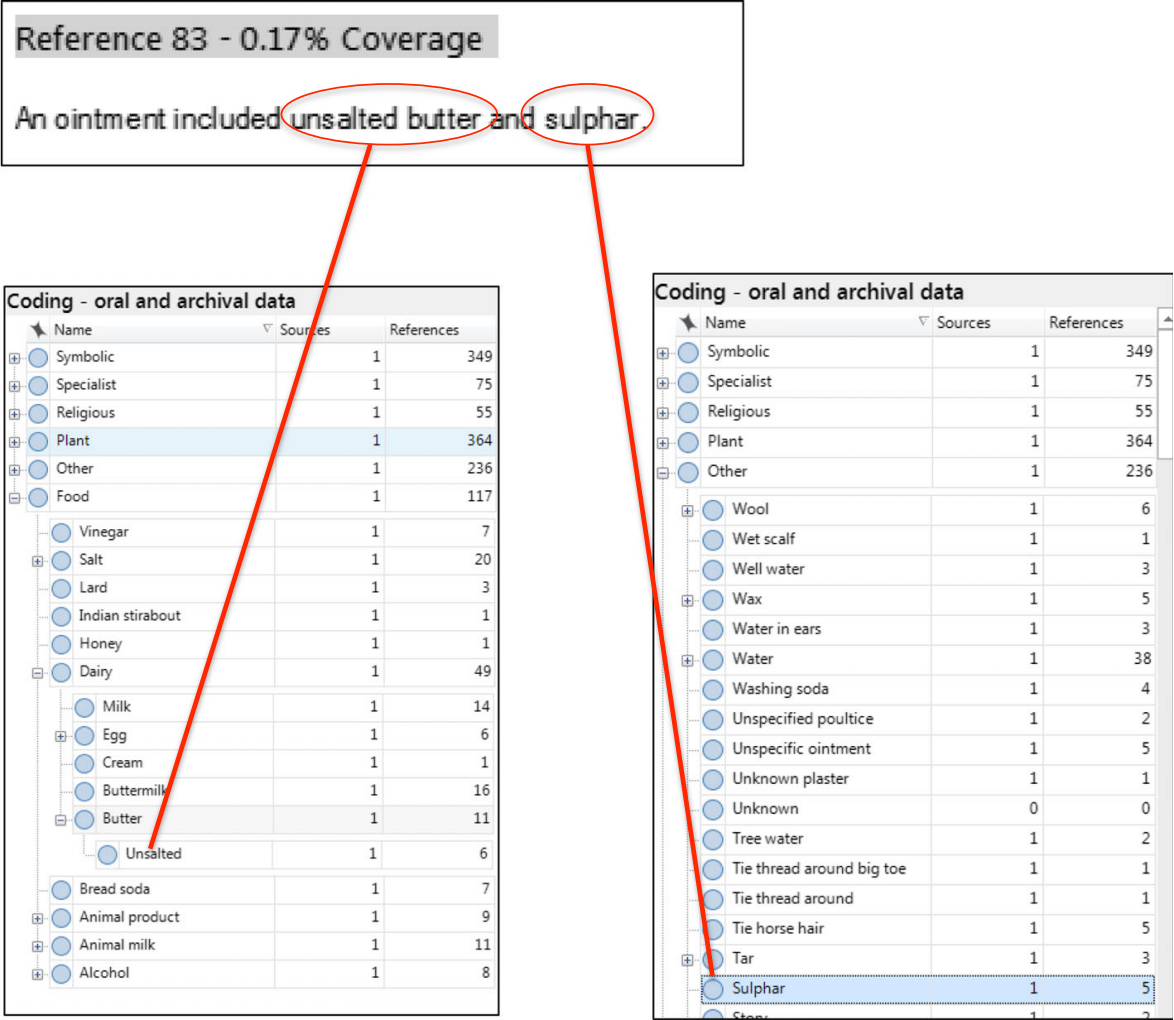
Example of NVivo coding for TMK data entry, nodes and sub-nodes

All the TMK data was collated and imported into the qualitative analysis software NVivo. Based on ‘grounded theory’ as first described by Glaser and Strauss [36] and content analysis as defined by Krippendorff [37], the data was not grouped according to pre-defined categories, rather each TMK data entry was disseminated into different parts (nodes), also described as ‘incidents’ [36] or ‘units’ [38]. Open coding was carried out on the dataset. Interesting features, substances and or methods described from each TMK entry were coded as nodes in a systematic way across the entire dataset. These nodes were allocated clear labels and definitions to serve as rules for inclusion. Figure 2 illustrates how one TMK entry was filed under relevant nodes.

Sub-nodes were also created to record additional detail, i.e. ‘the root of a dandelion’ would be filed under the node ‘dandelion’ and attached sub-node ‘root’. All the related ‘nodes’ were grouped, forming the structure for treatment categories. The main categories identified were plant, symbolic, religious, food, specialist and other. The nature of the analysis resulted in single TMK entries featuring in multiple categories.

## Results and discussion

### Classification of traditional knowledge in the Schools’ Manuscript Collection

**Fig. 3:**
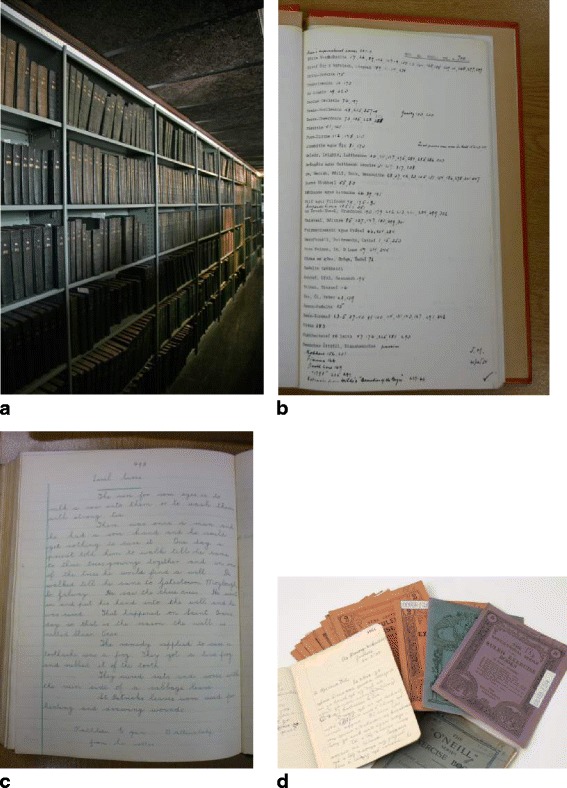
**a** Large manuscripts. **b** Index for LM. **c** Sample entry. **d** Small copybooks

All of the schools involved in the Irish Schools’ Folklore Scheme forwarded the material collected by the children to the National Folklore Collection. The materials archived included the pupils’ small copybooks (SC) and the large manuscript (LM) of selected material from each individual school (Fig. 3). For the purpose of this study and for more extensive analysis, the SMC material is referred to as traditional knowledge (TK). The specific TK topics documented in the LM were identified through the index system generated by the National Folklore Collection (Fig. 3b).

### Arranging the traditional knowledge topics into themes

**Fig. 4:**
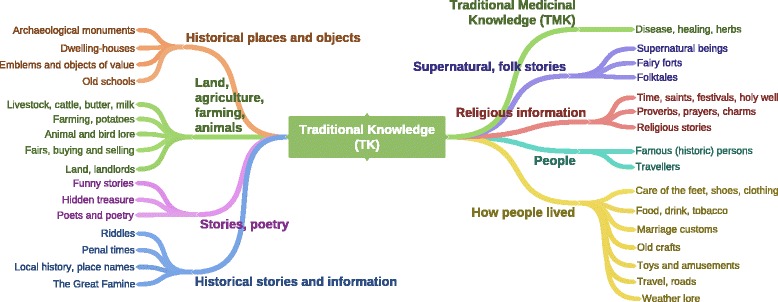
TK topics indexed in the LM material collated into nine TK categories

For this study, all the TK topics listed in the index, from the nine schools’ material, were collated and then organised into broader themes (Fig. 4). The LM volumes, corresponding to the participants in this study, contained a total of 23 topics that are indexed in the archive as TK, covering a range of subjects such as poets and poetry, the penal times, proverbs, prayers and charms, old crafts, weather lore etc. The TK categories used in the archive were analysed in this study and further collated into nine broader categories. One of the TK categories generated is traditional medicinal knowledge (TMK) and includes the material in the LM that was indexed under ‘disease, healing and herbs’. As there was no index system for the SC, all the corresponding SC entries were examined for ‘disease, healing and herbs’ references, and this was transcribed and classified as TMK and is presented herein.

### Further classification of traditional medicinal knowledge

**Fig. 5:**
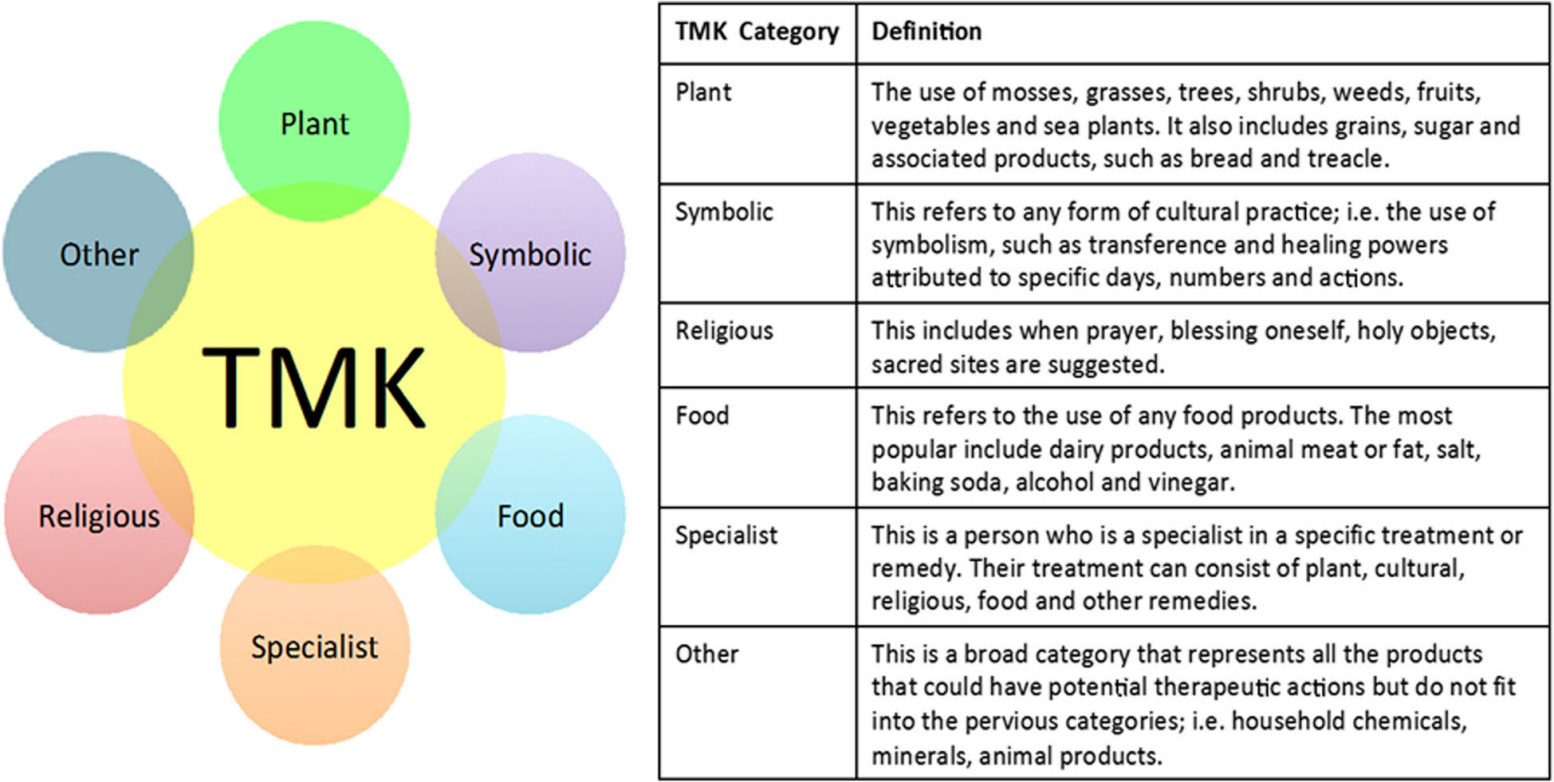
Classification of tradition medicinal knowledge categories used in this study, established using NVivo

The TMK material in this study has been analysed and collated utilising the analytical software NVivo, as described above under the ‘Statistical analysis’ section. The main categories that have been generated are: plant, symbolic, food, specialist and other (Fig. 5).

### Impact of the Schools’ Folklore Scheme on the respondents

The majority of participants (82%) believed the scheme had a positive impact on them, two (18%) stated it did not have any significant impact and no one thought it had a negative impact. All 11 believed that a comparable scheme would benefit children today. When asked if the scheme had an impact on their relationship with Irish culture, eight (72%) felt it had a positive impact. The participant living in the US responded, ‘Yes, absolutely, even to this day. It brings back my thoughts of how good Irish culture was, and that I made a promise to the Statue of Liberty when I landed in New York that I would try to keep Irish culture going here’. Other replies from interviewees include ‘it was very worthwhile, it started and interest in me’, ‘it gave a great understanding of it’ and ‘it made me value it’. Only one participant (9%) had no opinion, and two (18%) stated it did not result in a positive impact with Irish culture. Also, eight (72%) of the participants stated that they enjoyed taking part in the scheme.

### Oral recollections of the teachers, their impact on material collected and potential bias

Questions concerning the gender of the teacher and religious ethos of school were included in the survey. Six of the participants had a male teacher and five had a female teacher. One of the teachers was a priest, and one was a participant’s mother. All of their schools were Catholic, i.e. no religious comparison was made. Detailed information from the participants, relating to their memories concerning their teachers and of their contributions to the scheme, were collected (Table 2). The participants’ unique recollection of their teachers’ involvement and their teachers’ instruction imply there may have been a level of bias in how information was gathered and collated. Interviewees commented that their ‘teachers were very committed’ to teaching and believed the standard of teaching was ‘very high’ when they were children. One participant ‘loved school and loved Mrs. Neary’, who she believed was ‘a great teacher and very versatile’*.* Each participating teacher was in control of what topics the children collected information on, and which material was subsequently transcribed into the large manuscripts. The teachers’ perceived understanding of the scheme, their own personal interests and their enthusiasm towards the scheme impacted on the material collected and documented in the SC and the SMC. Three of the participants stated that their teacher showed a specific interest in the scheme. One recalled how her teacher was ‘very much into it’ and stated ‘our teacher was very interested and that and we used to love tell him stories’. Another participant was the daughter of a teacher that partook in the collection of material. She recalled her mother being particularly dedicated to the scheme and stated ‘she was very enthusiastic’ about it, and that ‘she loved Irish’. Although the scheme intended the children collect the material, her mother also collected information. One of her mother’s TMK entries in the SMC is to ‘give a large saucer of milk to a ferret and give the leavings to a child who has the whooping cough. It will soon get better’. She recalls accompanying her mother travel long distances to different homes in the evenings to document peoples’ stories for the scheme. In contrast, one participant believed his teacher did not have much interest in the scheme, and as a result, ‘the kids had not a clue what it was about at all’.

### Oral recollections of TMK collected for the scheme

For this study, the oral TMK recollections of the original participants have been linked to two particular aspects, the individuals’ personal TMK contributions located in the SC as well as any referenced TMK contributions in the LM. Additionally, looking at the overall TMK content in the corresponding schools’ allows us to observe correlations and establish context for TMK practice in relation to the time period and regional differences. The participants’ recollections of TMK were gathered by asking questions regarding ‘health practices, local cures and remedies’ that they could recall from their childhood and their participation in the scheme. The interviews were open-ended. The narrative was relatively broad and qualitative and allowed for depth, nuance and variety in responses. In the interviews, nonverbal communications were also observed. The participants ranged in age from 86 to 93 years. There was a discernible difference in their level of recall and mental clarity, although all were comfortable with participation in the study and very enthusiastic to have some value and relevance in a modern study. None of the participants indicated that they were aware of the historical and social significance of the scheme. One participant stated that she wasn’t even aware she was taking part in a ‘scheme’ as such and believed what they were collecting the information for ‘homework’.

The topics that the participants recalled collecting information on were varied, including ‘how people lived’, ‘historical ruins’, ‘old superstitions’, ‘local burial grounds’, ‘ghost stories’, ‘fairies’, ‘how they did jobs’ and ‘traditional cures’*.* Only five of the participants stated they recorded TMK for the scheme. However, of the six participants that stated they did not record TMK for the scheme, five of these stated they recalled TMK being used at that time but were not asked and or did not provide this information for the purposes of the scheme. None of the five participants that did record TMK stated they passed this TMK information on to younger generations. In relation to passing on TMK, one participant responded ‘Oh I don’t think anyone uses any of them now, it’s off to the chemist’. Another participant described people’s use and belief in TMK at that time, by stating that ‘it worked for them and they had the belief in it. There was nothing else, some of the things did really work’. When asked if they passed on any of the general TK information collected to younger generations, three stated that they did and seven stated they did not pass on any of the information. One participant described how she passed on the information to her children and her grandchildren and stated they were ‘all interested’ and ‘all into it’. Another participant stated she did not pass on any information as ‘I do not think I did because you see when I was rearing my family there were very good children’s books on the market and I would read to mine at night’.

### Direct links with corresponding archival material relating to TMK

Many of the entries in the SMC are not referenced to specific pupils, and in fact, the material corresponding to participant 1 (P1) (Swords National School, County Dublin) was not located within the archive. LM material was located for the eight schools corresponding to the ten remaining participants. Corresponding bundles of small copybooks (SC) were located for six of these eight schools (Fig. 3d). Within these bundles, copybooks belonging to four participants were found and three of these (P3, P8 and P9), contain TMK.

In his interview, participant 3 (P3), who attended St. Columba’s Abbey in County Meath, recalled six practices of TMK. These consist of two treatments using the ‘dandelion’ plant to get rid of ‘warts’ by ‘using the milk from the stem’ and a general treatment of ‘making a drink out of dandelion roots’ for no specific ailment. The remaining four include a combination of the treatment categories: ‘symbolic’, ‘religious’, ‘other’ and ‘specialist’ as defined in Fig. 5. These were elaborated in the interview as ‘going to a specific lady’ and visiting ‘Knock, a religious site’ to treat ‘warts’, ‘prodding your eye with a gooseberry thorn’ to treat a ‘sty’ and ‘yellow clay and (water from?) a holy well’ to treat a ‘sprain’. His school’s LM material, his own SC and 18 other SC were located. Within these sources, there are 89 TMK entries in the LM material and 158 TMK entries in the SC. P3’s personal copybook contains ten TMK entries. Two of these entries correspond directly to this participant’s recollections of ‘yellow clay’, ‘a Holy well’ and the ‘gooseberry thorn’. There is a direct reference to P3 in his school’s LM material. There are two citations but neither corresponds with either the participant’s oral recollection or his SC material. Both of these entries are ‘plant’ based and consist of using ‘a potato’ to treat a ‘wart’ and to ‘boil and strain celery seed and drink’ for ‘rheumatism’. Interestingly, there are two entries in the SMC that are referenced to another pupil but correspond with the TMK recalled by P3. They include using ‘the juice of the dandelion’ to treat ‘liver trouble’ and pointing ‘gooseberry stalks’ to treat a ‘sty’. His recollections show some correlation with what is documented in his corresponding SC. Although there is no correlation with what is documented under his name in the LM, there is evidence of his recalled TMK referenced under another pupil’s name. This could be attributable to the common use of dandelion in Irish herbal medicine. Participant 8 (P8), who attended Ardagh National School in County Mayo, recalled four pieces of TMK. These consist of a ‘food’ treatment recommending ‘fat bacon for a corn’, ‘other’ treatments ‘rock water for warts’, ‘a poultice for an infection’ and a ‘specific person’ and ‘symbolic’ treatment stating ‘her grandmother had a charm to cure’. Her school’s SMC material, her own small copybook and 15 other SC were located. Within these sources, there are 46 TMK entries in the SMC and 109 TMK entries in the SC. P8’s own copybook contains eight TMK entries. None of these entries correspond with P8’s recollections. There are no entries referenced to P8 in the LM.

Participant 9 (P9) attended Killacorraun National School in County Mayo and recalled eight pieces of TMK. These include three ‘plant’ treatments: to ‘roast an onion on the fire and place onto the ear for an earache’, to ‘rub a briar on a hive’ and to treat a ‘burn’ by making ‘an ointment out of the root of the comfrey plant’. They also include the ‘symbolic’ and ‘specialist’ treatments: ‘to pick the first blackberrys that come out and put them under a stone and as they rot the warts will dissolve’, to get the cure for ‘thrush’ from ‘a person whom never met their father’ and to ‘go to a specific person on a Tuesday and a Thursday for three weeks to cure a headache’*.* The subsequent two treatments are ‘food’ based and involve boiling ‘buttermilk and oaten meal to make a paste for boils’ and drinking ‘the milk of a female ass for whooping cough’. This school’s SMC material, two small copybooks belonging to P9 and nine other SC were located. Within these sources, there are 35 TMK entries in the SMC and 48 TMK entries in the SC. P9’s copybooks contain 14 TMK entries. Three of these entries correspond to her recollections, using ‘comfrey’ to treat ‘burns or sores’, ‘get a child that never saw its father to blow its breath in to the mouth of the person three times’ to treat ‘thrush’ and ‘drink asses milk’ to cure ‘whooping cough’. There are no references to P9 in the SMC. However, there is very little referencing in this school’s material and four TMK entries in this school’s SMC material that are not referenced with a specific pupil’s name to correspond with her recollections. These TMK entries recommend: a person to go to a ‘seventh son’ for ‘headaches’, ‘a person who never saw his father’ has the cure for ‘thrush’ and drink ‘an asses milk’ to treat ‘whooping cough’. The final corresponding treatment is a variation of P9’s recollection to treat ‘hives’. Instead of ‘rubbing the briar’ on the affected part, the version in the SMC recommends crawling ‘three times under a briar with both ends of it stuck in the ground’.

It is unfortunate that only four of the participants’ corresponding SC were located, and of these copybooks, only three contain TMK. A correlation between the oral recollections and the written was identified for two of the participants. However, in general, there is an inconsistency between the oral and written. This may result from the clarity of memory related to their age and the 80-year time gap between taking part in the scheme and carrying out the interviews. Furthermore, there are also discrepancies between the SC and SMC material, which may result from approaches taken by or attitudes of teacher (toward a particular, child, family, content or handwriting).

### Oral TMK recollections corresponding to school’s overall TMK material

The remaining participants (P1, P2, P4, P5, P6, P7, P10 and P11) have no personal TMK references in either the SC or LM. Their oral TMK recollections varied in clarity, content and enthusiasm. There specific TMK comments have been compared with the overall TMK content in their corresponding schools’ SC and LM material.

P1 attended Swords National School in County Dublin. She stated that she did not record any TMK information for the scheme. However, she did mention ‘if you got the sting of a nettle you wound a dock leaf around it’, and she told a story of a neighbour who had a ‘secret recipe for all ills’ that was kept in a special ‘chamber pot’. Unfortunately, her corresponding school’s LM or SC material was not located in the archives. Participant 2 (P2) who attended Keenagh National School in County Mayo recalled ten practices of TMK that she collected for the scheme. Her school’s LM material contains no TMK entries. However, one of the sources that she named in her interview ‘Peter Gilroy’ is referenced as an informant in a neighbouring school’s LM material. This highlights that within small communities, children from different schools may have sourced information from the same people. In addition, it is well known that medicinal knowledge and practices were often ‘hereditary’ and associated with certain families [39]. Participants 4 and 7 (P4 and P7) attended Belmullet National School in County Mayo. P4 did not recall any TMK and P7 recalled four TMK practices. These include a ‘plant’ treatment of ‘carrageen moss made into a drink called lamoge for a chronic cough’, ‘food’ and ‘other’ treatments of ‘goose grease and flannel was put on the chest’ for a ‘bad chest’, ‘poultices were commonly used for a stone bruise’ and ‘beating the white of an egg, sugar, hot milk and butter to make a drink for a sore throat, a cough or bronchitis’. This school’s LM and ten SC were located. Within these sources, there are six TMK entries in the LM and there are no TMK entries in the SC. None of the TMK recalled by P7 corresponds with the entries in the LM, and furthermore, none of the six treatments in the SMC related to either ‘plant’ or ‘food’ treatment categories. They all consist of practices that are of a ‘symbolic’ and or involve a ‘specialist’.

Participant (P5) attended Ballinlas National School in County Galway. She recalled one piece of TMK that involved ‘a neighbour’ whom had ‘the cure for the burn. It was a bottle made up with a secret recipe’. Her school’s LM material was located and contains 15 TMK entries. None of the entries relate to her recalled piece of TMK information. The TMK entries in her school’s LM material consist of a combination of all five ‘treatment categories’ with seven recommending the use of a ‘plant’, i.e. ‘a cabbage leaf’ for ‘cuts and sores’, ‘St. Patricks leaves were used for healing and drawing wounds’, ‘herbs that grew in a field’ were ‘used for fits’, ‘a bile was cured by soap and sugar’, ‘strong tea’ externally to treat ‘sore eyes’, ‘boiled flaxseed mixed with vinegar or lemon juice, sugar and treacle’ to treat ‘coughs and colds’ and a ‘cure for a toothache is to boil nettles in the month of March and drink the juice three times during the year’. Participant 6 (P6) attended Camphire Primary School in County Waterford. She did not recall collecting or submitting TMK information for the scheme; however, she did state that ‘we used millions of home remedies’. She specifically mentioned using ‘the inside of an egg for a sore’, ‘putting a sock around your neck for a sore throat’, ‘a dock leaf for a nettle sting’ and to treat a sore leg, ‘there was a woman you had to visit three times and she plastered it up’. Her school’s LM material was located and contains 25 TMK entries. Interestingly, none of these TMK entries included a ‘plant’ treatment. Participants 10 and 11 (P10 and P11) attended Kilchreest National School in County Galway. P10 did not recall any TMK and P11 recalled five TMK practices. These are all ‘plant’ treatments and recommend ‘dandelion for anaemia’, ‘red rose for a sore eye’, ‘ivy leaves for open sores’, ‘blackcurrant jam for hoarseness/sore throat/cough or colds’ and ‘meadowsweet for stomach problems’. This school’s LM material and 19 SC were located. Within these, there are 17 TMK entries in the LM and six TMK entries in the SC. None are referenced to either P10 or P11. None of P11’s recollections correspond with either TMK in the SC or LM, and furthermore, none of the TMK entries from this school documented in the SC correspond with the TMK entries in the SMC.

### Plant traditional medicinal knowledge

The TMK information from both the participants’ oral recollections and the SMC is comprised of a combination of the treatment categories (Fig. 5). For the purpose of this paper, the ethnopharmacological ‘plant’ TMK information has been further examined from two positions. Firstly, all of the plant-related treatments recalled in the interviews and the participants’ personal contributions within the archives have been analysed (Additional file 1). Secondly, the plant treatments gathered from the interviews have been collated and analysed together with the total content of their corresponding schools’ archival material (Additional file 2).

### ‘Plant’ TMK from the oral and personal contributions located in the archives

The oral TMK practices and the participants’ personal entries in the archives combine to a total of 76 (100%) TMK entries. Of these, 25 (33%) include the use of a plant and there are 18 plant species cited (Additional file 1). There is a combination of native, introduced and imported food products within these.

### Total ‘plant’ entries from oral and corresponding schools’ SMC material

When all the oral and schools’ sources are combined, there are a total of 596 (100%) TMK entries. Of these, 174 (29.19%) include the use of a ‘plant’. Some of the TMK entries include multiple plant species; thus, there are 191 ‘plant’ mentions and 64 different plant species cited. The list of the most frequently cited plant species from combined sources is presented in Additional file 2.

### Plants directly linked to participants

Dandelion (*Taraxacum officinale*) is mentioned three times, by two participants. The white sap from the stem is recommended for external use on warts, and the roots boiled in water can be taken internally for anaemia. It is also one of the most highly cited species among all the TMK entries examined (4.2% of all ‘plant mentions’), and it is documented as a treatment for gastrointestinal and musculoskeletal conditions (Additional files 1 and 2). *T. officinale* is abundant throughout Ireland, and the literature supports its medicinal use [24, 35, 40–43]. There is extensive evidence of the global use of this medicinal species throughout history, e.g. in tenth- and eleventh-century Arabian medicine [44]; in the sixteenth century, the German physician and botanist Leonhard Fuchs documented its use for gout, diarrhoea, blister, spleen and liver complaints [45]. In 1653, Culpeper also recommended the roots and leaves for the liver and spleen. Its use has also been recorded in North America, China, Turkey and Mexico [45]. The European Medicine Agency recognises *T. officinale* root and herb, based on ‘well-established use’ as a treatment for digestive and urinary complaints [46]. The majority of research conducted on *T. officinale* has focused on the leaves, roots and flowers [44]. Huber et al. recently identified phenolic inositol esters, triterpene acetates and the sesquiterpene lactone taraxinic acid β-d-glucopyranosyl ester in the root latex [47]. The phytochemical composition of *T. officinale* and the pharmacological properties of its metabolites including the antimicrobial and anti-inflammatory effects of its sesquiterpene lactones, the reduction in cholesterol absorption by its phytosterols and the presence of immunomodulatory flavonoids and effects of coumarins on cardiovascular disease [45, 48], would support the reported use in Ireland as a tonic. In 1998, Rudenskaya et al. [49] identified serine protease activity in the latex of *T. officinale* roots. Serine protease modulators in the sap may be linked to the traditional use of *T. officinale* in the treatment of warts, although further research is required on stem sap [50].

Comfrey (*Symphytum officinale*) is the only plant that directly correlates from the oral to a participant’s written entry in the SMC. P9’s oral and archival entries recommend external application of an ointment made from the root of the plant to treat burns and sores. *S. officinale* is also one of the most highly cited plant species overall (2.1% of all ‘plant mentions’). The root is documented as a treatment for burns and sores in both humans and animals and the plant is recommended as feed for pigs (Tables 2 and 3). These entries correlate with previous accounts of external dermatological and fodder use in Ireland from Moloney [40], Allen and Hatfield [24] and Wyse Jackson [35]. Pliny the Elder’s (23–79 AD) encyclopaedia *Natural Historia* recommended the root as a treatment for bruises and sprains and it is similarly cited in Dioscorides’ *Materia Medica* (50–70 AD) [51] In 1653, Culpeper added to its indications to include it as a treatment for rheumatism and gout and stated the leaves could be used, although they are not as effective as the roots [52]. In more recent times, the European Committee on Herbal Medicinal Products (HPMC) has approved the use of *Symphytum officinale* L., root for the treatment of ‘bruises and sprains’, based on ‘well-established use’ [53] The molecular mechanism of action of *S. officinale* root has not been completely elucidated. However, allantoin and rosmarinic acid are the most likely bioactive compounds contributing to dermatological healing actions [54]. Allantoin increases the smoothness of the skin, promotes cell proliferation and wound healing by stimulating the metabolic process in the subcutaneous tissue and stimulating cell growth, resulting in epithelialisation and a protective effect on the skin. Rosmarinic acid possesses an anti-inflammatory action among others [55].

**Table 3:** Participant recollections

Participants	Specific interest shown by teacher	Where the participant obtained the information that they recorded in the SFS	How long the participant believes the SFS went on for	Recalled recording TMK in the SFS	Did not record TMK in the SMC but stated they knew TMK used at that time	Passed on information to younger generations	Overall impact of the SFS
P1	–	Father	Once off	No	Yes	Yes	Positive
P2	No	Neighbours and parents	One term	Yes	–	No	Positive
P3	No	Mrs. Kenny	One year	Yes	–	No—but his daughter was present and said yes	Neutral
P4	Yes	Father and neighbours	More than a once off, for a while	No	Yes	No	Positive
P5	–	Grandmother and neighbours	–	No	Yes	Yes	Does not recall
P6	–	Neighbours	A long time but less than a year	No	Yes	Yes—informed by her daughter	Positive
P7	Yes	Grandparents, parents, and people in the community	Every night for a month	No	Yes	No	Positive
P8	Yes	Grandmother and father	Two years	Yes	–	Did not say	Positive
P9	–	Old people living in the village	A year or two—weekly	Yes	–	No	Positive
P10	–	Father	On and off	No	No	No	Positive
P11	–	Father and neighbours that visited the house	–	Yes	–	No	Positive

Dock (*Rumex* species) leaf is mentioned orally by two of the participants as a treatment for a sting from a ‘nettle’ (*Urtica diocia* L.). Within the overall data, the *Rumex* spp. is cited eight further times (5.2% of all ‘plant mentions’). All the entries recommend the same treatment for a sting, although one suggests ‘chewing the leaf’ before applying. The entries cite a range of vernacular names to describe this species, including: ‘dock’, ‘docken’, ‘capog’, ‘cappoc’ and ‘copog’. There are a number of different *Rumex* species in Ireland referred to as ‘dock’. However, *Rumex obtusifolius* is widely known for the ability to ease the sting of a nettle [35]. The irritation by a nettle sting is considered to be both biochemical and mechanical. The stem and the underside of the leaves of the *U. diocia* are covered in small spicule-type hairs. The biochemical reaction is believed to be the result of formic acid, histamine, serotonin and acetylcholine that is found in the hairs and nettle fluid [56]. The mechanical reaction is believed to be the impalement of spicules into the skin [57]. *R. obtusifolius* leaf contains the flavonoids procyanidin B1, B2, B3, B7 and gallate [58] and has demonstrated antioxidant and antimicrobial actions [59]. *Rumex* spp*.* is indigenous to Europe. *R. obtusifolius* and has been introduced to many other parts of the world, including: Africa, North and South America [60].

Potato (*Solanum tuberosum*) is renowned as the ‘archetypal Irish vegetable’. *S. tuberosum* is a domesticated hybrid plant that was introduced to Europe and Ireland from South America in the sixteenth century. It has had a profound impact on Irish culture, landscape, history and economy [35]. One participant described how to boil and mash a potato and place around one’s neck to treat a sore throat. Another participant’s entry in the SMC describes how one should ‘rub a potato on a wart’ for a number of consecutive days. There are a total of six entries (3.1% of all ‘plant’ mentions) that recommend *S. tuberosum* for dermatological, musculoskeletal and respiratory complaints (Additional files 1 and 2). The entries include external applications of both raw and heated *S. tuberosum*. Similar historical medicinal uses have been documented in Ireland, England, Wales and North America [5, 61, 62]. Glycoalkaloids present in the *Solanum* species have demonstrated antifungal, anti-allergic, antipyretic, anti-inflammatory, hyperglycaemic, antibiotic and anti-carcinogenic properties [63, 64]. In addition, α-solanine, α-solamargine and α-chaconine have shown antifungal and cytotoxicity activity [65, 66].

Carrageen moss (*Chondrus crispus*) is the only sea plant documented in this study. Sea plants have been used in Ireland for centuries, as a food, fodder, soil fertiliser and medicine, and they are considered to be especially valuable for their high iodine and mineral content [35]. P7’s description of her mother making a drink with *C. crispus* to treat a chronic cough corresponds with previous reports from Moloney [40], Jude [67], Logan [42], van Wyk and Wink [68], Allen and Hatfield [24], Dolan [5] and Pina et al. [69]. *C. crispus* grows abundantly along the rocky coasts of the north Atlantic and north Pacific oceans [69]. The mucilage present in this plant is believed to be responsible for soothing and anti-inflammatory properties that help alleviate respiratory conditions [70]. Various homemade drinks using *C. crispus* are still widely used today in Ireland for bronchial conditions [71].

Oats (*Avena sativa*) have been grown in Ireland for centuries as a food crop for both humans and animals [35]. One participant recalled an oatenmeal paste that was made to treat a boil, and an entry in the SMC details how oatgrass is a treatment for a ‘skinned foot’ on a horse. This external application of *A. sativa* correlates with a long history, since Roman times, of being used worldwide, as a treatment for a range of skin conditions [60–62]. ‘Colloidal oatmeal’ is a commercially available topical oat product that has been available since the 1940s. Chemical polymorphism is attributed to its ability to protect and sooth the skin [72]. *A. sativa* also possesses different types of phenols which exert an antioxidant and anti-inflammatory activity [73]. Wound healing and antioxidant activity demonstrated by *A. sativa* has been linked to the presence of avenanthramides, flavonoid-type compounds as well as vitamin E and phytic acid [74].

Meadowsweet (*Filipendula ulmaria*) is suggested for ‘stomach problems’ by one of the participants. This plant is native throughout most of Europe and western Asia and has been successfully introduced and naturalised to North America [75]. *F. ulmaria* was one of three plants considered to be most sacred by the Druids [70], and it has a long history of use in Europe as an anti-inflammatory, analgesic, diuretic and anti-rheumatic [35, 68, 70, 76]. The seventeenth century book *The Complete Herbal* recommends using the roots of the plant ‘made into a powder, and mixed with honey in the form of an electuary, doth much help them whose stomachs are swollen, dissolving and breaking the wind which was the cause thereof…’ [52]. However, use of the herb and flower are most frequently cited, and the European Medicine Agency has accepted it be sold as a ‘Traditional medicinal product for the supportive treatment of common cold, for the relief of minor articular pain’ based on ‘well-established use’ [76]. Both *F. ulmaria* aerial parts and roots have demonstrated anti-inflammatory activity in vitro and in vivo; although, the aerial parts exhibited a higher level of activity [77]. Salicylic acid and its derivatives are linked to its anti-inflammatory activity [77, 78].

There is one entry from P9 in the SMC that recommends St. Patrick’s Cabbage (*Saxifraga spathularis*) for ‘a sore lip or boil’. Five additional entries (3.1% of all ‘plant mentions’) suggest St. Patricks Cabbage for similar dermatological conditions (Additional files 1 and 2). Interestingly, there is no evidence of this plant being used medicinally. However, all the entries are from two schools in the same locality of County Mayo, so there is a possibility that the vernacular name is referring to an alternative plant or it is a local practice.

One participant recalled Red Rose (*Rosa* species) as a treatment for the eyes. Ireland is home to a wide variety of wild rose species. There are at least ten native roses known with many hybrids, thus identification is difficult. Many of the rose varieties in Ireland produce rosehips (fruits). These were a significant wild fruit in ancient Ireland and were often used to make jam, jelly, syrup, pie filling and wine [35]. Culpeper recommended a decoction of ‘red roses’ made with wine as a treatment for headache and pain in the eyes, ears, throat and gums and another treatment of rose leaves in a mixture with ‘beans’, ‘frankincense and the white of an egg’ to alleviate swollen eyes [52]. There have not been any specific studies into the use of *Rosa* spp. for eye conditions. However, the significant content of bioactive compounds present in rosehips is known to have a high level of antioxidant, antimicrobial, antifungal and anti-inflammatory activity [79]. These actions are specifically linked to polyphenols, vitamins C, E, B and carotenoids found in this species [35, 68, 79].

Castor oil (*Ricinus communis*) was orally mentioned by one participant as a treatment. However, they did not state for what condition. It is one of the most highly cited plants overall (2.6% of all ‘plant entries’) and includes three entries in the SC that recommend a mixture of castor oil and marigold to treat a burn and one entry that recommends a mixture of sulphur and castor oil to treat sourpock in cattle. The oil is obtained by pressing the seeds of the plant *R. communis.* The plant is believed to be native to eastern China and is now cultivated in hot climates around the world, specifically India and southern Asia. It has a long history of use, and in Roman times, it was known as the ‘Palma Christi’, as the leaves were thought to resemble the palm of Christ*.* The oil made from *R. communis* was traditionally taken internally as a laxative and purgative although this is no longer recommended due to its toxicity [80]. In the nineteenth century, a number of oils, including Castor oil, were recommended as the first aid for burns. At the time, the oils were suggested for two reasons: their ability to exclude air from the burn and because of their accessibility, quick application was possible [81, 82]. However, antioxidant activity has been demonstrated by methyl ricinoleate, ricinoleic acid, 12-octadecadienoic acid and methyl ester that are found in the seeds [80, 83] and the astringent and antimicrobial actions of tannins, flavonoids, triterpenoids and sesquiterpenes also found in the seeds provide wound healing abilities, by promoting wound contraction and increasing the rate of epithelialisation [80, 84].

Celery (*Apium graveolens*) is also one of the most highly cited plants. It is documented six times (3.1% of all ‘plant mentions’) for the treatment of rheumatism. The entries include recommendations to ‘boil the celery seed and drink’, ‘make a drink from celery’ and ‘eat celery’ (Additional files 1 and 2). It is found throughout Europe and in temperate and sub-tropical parts of Africa and Asia [85]. It is widely documented as used for rheumatic conditions, in Ireland and globally [24, 35, 86]. The stems are consumed worldwide as a food, and the leaves, seeds and essential oil are used medicinally. Its value as a traditional medicine is attributed to the presence of carbohydrates; flavonoids; alkaloids; and steroids; the compounds limonene, selinene, frocoumarin glycosides and vitamins A and C [85]. It is suggested that the medicinal effect of the seed on rheumatic conditions is due to its diuretic action [87].

Chickweed (*Stellaria media*) is an extremely common plant found throughout Ireland. There are four entries in total (2.1% of all ‘plant mentions’) recommending its use for dermatological and respiratory conditions (Additional files 1 and 2). The plant is also found throughout Europe and North America [88]. It has a history of treating a range of conditions including: skin diseases, burns and bruises, inflammation of the digestive, renal and respiratory tract, and rheumatism and joint diseases [68, 88–92]. In Irish mythology, the Celtic God Dian Cécht is believed to have recommended a combination of ‘chickweed, hazel buds, dandelions and wood sorrel to be boiled with oatmeal and taken in the morning and evening’ to treat ‘colds, phlegm, sore throat and the presence of evil things in the body, such as worms’ [93]. Culpeper also recommended the external application for a variety of ‘hot’ inflammatory conditions [52]. It is most commonly known as an external treatment for cuts, wounds, irritation and inflammation, as well as an internal remedy for rheumatism, respiratory complaints and jaundice [24, 35, 68, 87]. Saponin glycosides, coumarins and hydroxycoumaris, flavonoids, carboxylic acids, triterpenoids and vitamin C present in *S. media* contribute to anti-inflammatory action [88].

One of the participants recalled ivy (*Hedera helix*) leaves being used for open sores, and two further entries in the SMC (1.6% of all ‘plant mentions’) recommend it as a treatment for corns (Additional files 1 and 2). This corresponds with accounts listed by Culpeper [52], Wyse Jackson [35] from Moloney [40], K’Eogh [94], MacMahon [95] and Allen and Hatfield [24]. *Hedera* fossils have been recorded in Korea and Europe from approximately 5.8 million years ago, and *H. helix* is reported as used medicinally since Greek and Roman times. In present times, *H. helix* folium is indicated as an expectorant in the case of productive cough, based on ‘well-established use’ by the European Medicine Agency [96], and it is most commonly used in Germany [97]. The chemical components flavonoid rutin, quercetin, kaempferol and apigenin have demonstrated anti-inflammatory, antioxidant and vasoprotective properties. In addition, alpha-hederin, hederacoside B and C and hederagenin, triterpenoid saponins are attributed to the activity of the plant [98].

Tea (*Camellia sinensis*) is Ireland’s most popular and widely consumed beverage. One of the participants recalled a cold tea compress being used, but did not specify for what condition, and there are ten more entries in the SMC (5.8% of all ‘plant mentions’) recommending tea. These include using tea as a treatment for headaches and to bathe sore eyes with cold tea. One interesting treatment documented recommends one to ‘go to a wedding and drink the tea left by the bride and groom’ to cure whooping cough. *C. sinensis* origins are Asian. The earliest reference to tea goes back to more than 2500 years in China. Black tea arrived in Ireland in the early 1800s as an expensive and luxurious beverage for the upper and middle classes and later spread to become a common and popular drink for the rural and lower classes in the mid-1800s [35]. Similar reports of medicinal are documented in India, Kenya and Guatemala [99]. The chemical components of *C. sinensis* include polyphenols (catechins and flavonoids), alkaloids (caffeine, theobromine, theophylline, etc.), volatile oils and primary metabolites polysaccharides, amino acids and lipids. In order to produce black tea, the *C. sinensis* leaves undergo fermentation, resulting in an increased level of flavonoids and more concentrated amount of caffeine. Catechins and flavonoid compounds are reported to have antioxidant and antimicrobial properties, caffeine is linked to a diuretic effect and the astringent properties of tannis are believed to alleviate gastrointestinal conditions [100].

One participant recalled in her interview how an onion (*Allium cepa*) should be heated ‘on the fire and then [pressed] against the ear’ to treat an earache. One of the entries in the SMC corresponds with this, and two further entries recommend the juice of an onion for a wasp sting (2.1% of all ‘plant entries’) (Additional files 1 and 2). Ethnomedicinal use in Ireland corresponds with previous reports by Dolan [5] and Allen and Hatfield [24]. The *Allium* species was one of the first cultivated plant species used for culinary and medicinal purposes. It has been used medicinally worldwide for centuries. Culpeper [52] states ‘the juice of onions is good for either scalding or burning by the fire…and dropped in the ears, eases pains and noise in them’. It is described as a traditional remedy for a variety of respiratory ailments, bacterial infections, dysentery, ulcer wounds, scars, keloids, pain and swelling after insect stings [101]. *A. cepa* is reported to possess a range of actions, including anti-inflammatory and antioxidant [102] anti-genotoxic and anti-proliferative [103]. It is a rich source of phytonutrients, containing phenolic compounds such as flavonoids, saponins and sulphur compounds [104].

## Conclusion

The information captured in the SMC gives us an insight into the medicinal practices and beliefs of people in 1930s Ireland. The information is unique in terms of how, when it and why it was collected. While there are several similar studies, in terms of its scope and ambitions, there is no comparative body of ethnographic information globally. Contacting original participants offers a novel approach of looking at this material. It has provided us with a unique first-hand account of this historical initiative, an insight into how the scheme was implemented, how it impacted upon the children and captured viewpoints on perceived bias of teachers in the selection of content. We located 10 of the 11 participants’ schools contribution in the SMC. Focusing on ethnomedicinal ‘plant’ treatments, we were able to draw a comparison between the oral recollections and the archived material. Even though the sample size is small, this study is relevant since it offers a unique snapshot into how ‘knowledge about Irish medicinal plants was constructed’ and that such a study, in fact, did impact onto the lives of those who participated. While the cultural-political impact of the survey cannot be assessed, an underlying purpose had been to get people to identify with ‘Irishness’, and the survey may well have contributed to this.

We developed a novel approach using oral history as a tool both to capture the remaining informants’ perspective, but also to identify datasets in the Schools’ Manuscript Collection to be included in an analysis. We are, however, conscious that the situation in this case is unique, and that it will not be possible to repeat such a study in other contexts.

The plant species that were recalled by the participants and/or their personal entries in the archival material were identified, and the most highly cited plant species from all the data were collated. The species and their uses are common to recorded data throughout Ireland and are in keeping with key ethnomedicinal species used throughout the world. Chickweed, dandelion and oatmeal can be traced back to Irish mythology, and other species are found in ethnomedical systems throughout the world (e.g. the data reflects an oral identity that spans generations and that in many cases was not documented in writing until the 1930s). The oral identity reflected in the archival material is reinforced with the contributions of the respondents in this survey.

## Additional files

Additional file 1: Plant citing’s from participants’ oral recollections and their personal contributions documented in the SMC. It is a table of the plant species cited by participants’ and documented in the archives. It includes details on vernacular name, plant family, diseases treated and species origin. (PDF 30 kb)

Additional file 2: Most highly cited plant species from combined sources. It is a table that includes the most highly cited plant species overall, vernacular name, number of citations, diseases treated and species origin. (DOCX 19 kb)

## Abbreviations

LM: Large manuscripts
P1–P11: Participants 1–11
SC: Small copybooks
SFS: Schools’ Folklore scheme
SMC: Schools’ Manuscript Collection
TK: Traditional knowledge
TMK: Traditional medicinal knowledge
