# Supplementary material for: Are identities oral? Understanding ethnobotanical knowledge after Irish independence (1937–1939)
Source: J Ethnobiol Ethnomed. 2017 Nov 21;13:65. doi: 10.1186/s13002-017-0189-0 (PMC5699017; doi:10.1186/s13002-017-0189-0)
Supplement: Supplementary file 1 — Plant citing’s from participants’ oral recollections and their personal contributions documented in the SMC. It is a table of the plant species cited by participants’ and documented in the archives. It includes details on vernacular name, plant family, diseases treated and species origin. (PDF 30 kb) [file 13002_2017_189_MOESM1_ESM.pdf]

Family	Plant Species*	Vernacular name	No. of citations	Information source **	Disease Category***	Origin	Total no. of citations
Asteraceae	*Taraxacum officinale* (L.) Weber ex F.H.Wigg.	Dandelion	1<br>1<br>1	P11O<br>P3O<br>P3O	C<br>D<br>U	Native species	3
Boraginaceae	*Symphytum officinale* L.	Comfrey	1<br>1	P9SC<br>P9O	D<br>D	Possibly native or introduced species	2
Polygonaceae	*Rumex* species	Dock<br>Dock	1<br>1	P6O<br>P1O	D<br>D	Native species	2
Solanaceae	*Solanum tuberosum* L.	Potato<br>Potato	1<br>1	P2O<br>P3LM	R<br>D	Introduced species	2
Poaceae	*Triticum species* or *Avena sativa* L. or *Hordeum vulgare* L.	Bread	1	P2O	U	Possibly native or introduced species	1
Numerous families		Briar	1	P9O	D	Possibility of multiple species	1
Grossulariaceae	*Ribes nigrum* L.	Blackcurrant	1	P7O	R	Introduced species	1
Gigartinaceae	*Chondrus crispus* Stackh.	Carrageen	1	P7O	R	Native species	1
Euphorbiaceae	*Ricinus communis* L.	Castor oil	1	P2O	U	Imported product	1
Apiaceae	*Apium graveolens* L.	Celery	1	P3LM	M	Native species	1
Caryophyllaceae	*Stellaria media* (L.) Vill.	Chicken weed	1	P9SC	M	Native species	1
Araliaceae	*Hedera helix* L. or *Hedera hibernica* (G.Kirchn.) Carrière	Ivy	1	P11O	D	Native species	1
Rosaceae	*Filipendula ulmaria* (l.) Maxim.	Meadowsweet	1	P11O	G	Native species	1
Numerous families	(not defined culturally at the level of a botanical species)	Moss	1	P2O	UR	Possibility of multiple species	1
Poaceae	*Avena sativa* L.	Oatenmeal	1	P9O	D	Possibly native or introduced species	1
Amaryllidaceae	*Allium cepa* L.	Onion	1	P9O	R	Introduced species and imported food product	1
Rosaceae	*Rosa* species	Red Rose	1	P11O	E	Native species	1
Saxifragaceae	*Saxifraga spathularis* Brot.	St. Patricks Cabbage	1	P9SC	D	Native species	1
Amaranthaceae	*Beta vulgaris* L.	Sugar	1	P7O	R	Imported food product	1
Theaceae	*Camellia sinensis* (L.) Kuntze	Tea	1	P2O	U	Imported food product	1

*Here we give the most likely botanical species based on the common name of the taxon. Cleary, since these are based on historical documents no authentication is feasible. **P (1-11) = Participant (1-11); O = Oral recollection; SC = Personal contribution in the SC; LM = Personal contribution in the LM
*** C = Circulatory, D = Dermatological, E = Eye, G = Gastrointestinal, I = Infectious Disease, M= Musculoskeletal, N = Nervous system, R = Respiratory,

T = Teeth, U = Unknown or unspecified, UR = Urinary system, V = Veterinary
