# Supplementary material for: Are identities oral? Understanding ethnobotanical knowledge after Irish independence (1937–1939)
Source: J Ethnobiol Ethnomed. 2017 Nov 21;13:65. doi: 10.1186/s13002-017-0189-0 (PMC5699017; doi:10.1186/s13002-017-0189-0)
Supplement: Supplementary file 2 — Most highly cited plant species from combined sources. It is a table that includes the most highly cited plant species overall, vernacular name, number of citations, diseases treated and species origin. (DOCX 19 kb) [file 13002_2017_189_MOESM2_ESM.docx]

| **Family** | **Plant Species*** | **Vernacular name** | **No. of citations** | **Disease**  **category**** | **Origin** | **Total no. of citations** |
| --- | --- | --- | --- | --- | --- | --- |
| Poaceae | *Triticum species* or *Avena sativa* L. or Hordeum vulgare L. | Bread Bran Gruel | 9 3 1 | D,R,U R,V R | Possibly native or introduced species | 13 |
| Theaceae | *Camellia sinensis* (L.) Kuntze | Tea | 11 | D, E, N, R, U | Imported food product | 11 |
| Amaranthaceae | *Beta vulgaris* L. | Sugar | 10 | D, R, U | Imported food product |  |
|  |  | Treacle | 1 | R |  | 11 |
| Polygonaceae | *Rumex* species | Dock | 3 | D | Native species | 10 |
|  |  | Docken | 1 | D |  |  |
|  |  | Capog | 3 | D |  |  |
|  |  | Cappoc | 2 | D |  |  |
|  |  | Copog | 1 | D |  |  |
| Liliaceae | *Allium sativum* L. | Garlic | 9 | N, R, V | Introduced species | 9 |
| Asteraceae | *Taraxacum officinale* (L.) Weber ex F.H.Wigg. | Dandelion | 8 | C, D, G, M, U | Native species | 8 |
| Apiaceae | *Apium graveolens* L. | Celery | 6 | S | Native species | 6 |
| Oleaceae | *Olea europaea* L. | Olive oil | 6 | D, R | Imported food product | 6 |
| Solanaceae | *Solanum tuberosum* L. | Potato | 6 | D, M, R | Introduced species | 6 |
| Saxifragaceae | *Saxifraga spathularis* Brot. | St. Patricks Cabbage | 6 | D | Native species | 6 |
| Euphorbiaceae | *Ricinus communis* L. | Castor oil | 5 | D, U, V | Imported product | 5 |
| Urticaceae | *Urtica dioica* L. | Nettle | 5 | D, I, T, V | Native species | 5 |
| Caryophyllaceae | *Stellaria media* (L.) Vill. | Chicken weed | 4 | M, R | Native species | 4 |
| Fagaceae | *Quercus petraea* (Matt.) Liebl.  or *Quercus robur* L. | Oak | 4 | D, M | Native and introduced species | 4 |
| Amaryllidaceae | *Allium cepa* L. | Onion | 4 | D, R | Introduced species and imported food product | 4 |
| Boraginaceae | *Symphytum officinale* L. | Comfrey Comfry | 3 1 | D, V D | Possibly native or introduced species | 4 |
| Linaceae | *Linum usitatissimum* L. | Flaxseed | 3 | R | Introduced species | 3 |
| Araliaceae | *Hedera helix* L. or *Hedera hibernica* (G.Kirchn.) Carrière | Ivy | 3 | D | Native species | 3 |
| Malvaceae | *Althaea officinalis* L. | Marshmallow Marsh Mallows | 2 1 | S S | Possibly native or introduced species | 3 |
| Compositae | *Calendula officinalis* L. | Marigold | 3 | D | Native species | 3 |
| Numerous families | (not defined culturally at the level of a botanical species) | Moss | 3 | I, UR | Possibility of multiple species | 3 |
| Brassicaceae | Most likely a combination of *Brassica nigra* (L.) K.Koch, *Sinapis alba* L. and *Brassica juncea* (L.) Czern. | Mustard | 3 | T | Imported food product | 3 |
| Solanaceae | *Nicotiana tabacum* L. | Tobacco | 2 | D, T | Introduced and imported species | 3 |
| Betulaceae | *Alnus glutinosa* (L.) Gaertn. | Alder | 2 | C, I | Native species | 2 |
| Veronicaceae | *Digitalis purpurea* L. | Fox glove | 2 | R | Native species | 2 |
| Rutaceae | *Citrus limon* (L.) Osbeck | Lemon | 2 | R, U | Imported species | 2 |
| Compositae or Asteraceae | *Ambrosia* species or *Senecio* *jacobaea* L. | Ragweed | 2 | I | Introduced species or native species | 2 |
| Lamiaceae | *Lamium purpureum* L. | Red nettle | 2 | D, I | Native species | 2 |

*Here we give the most likely botanical species based on the common name of the taxon. Cleary, since these are based on historical documents no authentication is feasible. ** C = Circulatory, D = Dermatological, E = Eye, G = Gastrointestinal, I = Infectious Disease, M= Musculoskeletal, N = Nervous system, R = Respiratory, T = Teeth, U = Unknown or unspecified, UR = Urinary system, V = Veterinary
